# Ricochet Penetrating Head Injury: A Case Report and Brief Review of the Literature

**DOI:** 10.7759/cureus.101179

**Published:** 2026-01-09

**Authors:** Manar Alossaif, Sultan Alsalmi, Amenah Al Manasef, Majed Alomair

**Affiliations:** 1 College of Medicine and Surgery, Imam Abdulrahman Bin Faisal University, Dammam, SAU

**Keywords:** frontal bone, gunshot, head injury, intracranial gunshot wound, ricochet bullet, saudi arabia

## Abstract

Ricochet gunshot injuries to the head represent an uncommon form of penetrating cranial trauma requiring careful surgical decision-making. We report the case of a 28-year-old male who sustained a ricochet gunshot injury to the skull with an associated left-hand wound. He was transported to the emergency department by the Red Crescent and arrived hemodynamically stable, alert, and oriented, with a Glasgow Coma Scale score of 15/15. Surgical exploration and debridement were performed, and accessible bullet fragments were removed. The postoperative course was uneventful, and the patient was discharged in stable condition. This case highlights important neurosurgical considerations in the management of cranial ricochet injuries, particularly the balance between elevating the bone and preserving depressed fragments for potential tamponade. A brief review of the literature is included to contextualize management strategies and outcomes.

## Introduction

Civilian gunshot wounds to the head (GSWH) are a subtype of penetrating brain injury and are associated with a substantial burden of morbidity and mortality. This medical emergency carries a high fatality rate, with only approximately 10% of victims surviving long enough to reach the hospital. Among survivors, neurological deficits are frequently observed [1].

Ricochet projectile injuries constitute a distinct and uncommon mechanism of firearm injury, defined as "the continued flight of a rebounded projectile and/or major projectile fragments after a low-angle impact with a surface or object" [2]. Despite reduced velocity upon impact, ricochet bullets can still inflict substantial injuries and fatalities [3]. Nishshanka et al. [4] reported that most injuries result from misaimed bullets.

In the Kingdom of Saudi Arabia (KSA), firearm ownership is regulated through strict licensing laws. Although comprehensive national data on firearm injury incidence are not publicly available, existing evidence indicates that there are 82/100,000 firearm-related deaths in KSA. Additionally, accidental and unintentional firearm injuries account for approximately 24% of firearm-related deaths [5]. In recent decades, the Houthi-Saudi Arabian conflict has contributed to an increase in firearm-related injuries presenting to the Najran region in KSA, with a mortality rate reaching up to 7.9%, the majority of which are attributed to head injuries [6]. Firearm incidents often lead to prolonged hospitalizations, with an average stay of 15.45 ± 23.06 days (range: 1-150 days), reflecting their significant economic and social impact [7]. Head and chest injuries are among the most common and often fatal outcomes of firearm trauma in regions such as Dammam, KSA [8].

Ricochet bullets pose diagnostic and surgical challenges due to their unpredictable trajectories and potentially misleading external wounds [9]. Neurosurgical approaches to GSWH vary. Here, we present a rare case of a ricochet bullet penetrating the skull of a young male. We also review the literature to discuss the management dilemma of whether cranial bone elevation and reconstruction are necessary, or if preserving the fracture may serve as a natural tamponade to control hemorrhage.

## Case presentation

A 28-year-old male was transported by the Red Crescent after sustaining a gunshot wound to the skull in his apartment during a shooting incident. Paramedics found him conscious and ambulatory. Upon arrival in the emergency department, the patient was stable with vital signs. His airway was patent, and oxygen saturation was maintained. No obvious deformities were noted except for two scalp wounds and a first web space laceration in the left hand. Complete exposure during the primary survey revealed no evidence of other injuries. His Glasgow Coma Scale (GCS) score was 15/15. He was resuscitated with intravenous fluids and stabilized for further diagnostic workup.

**Figure 1:**
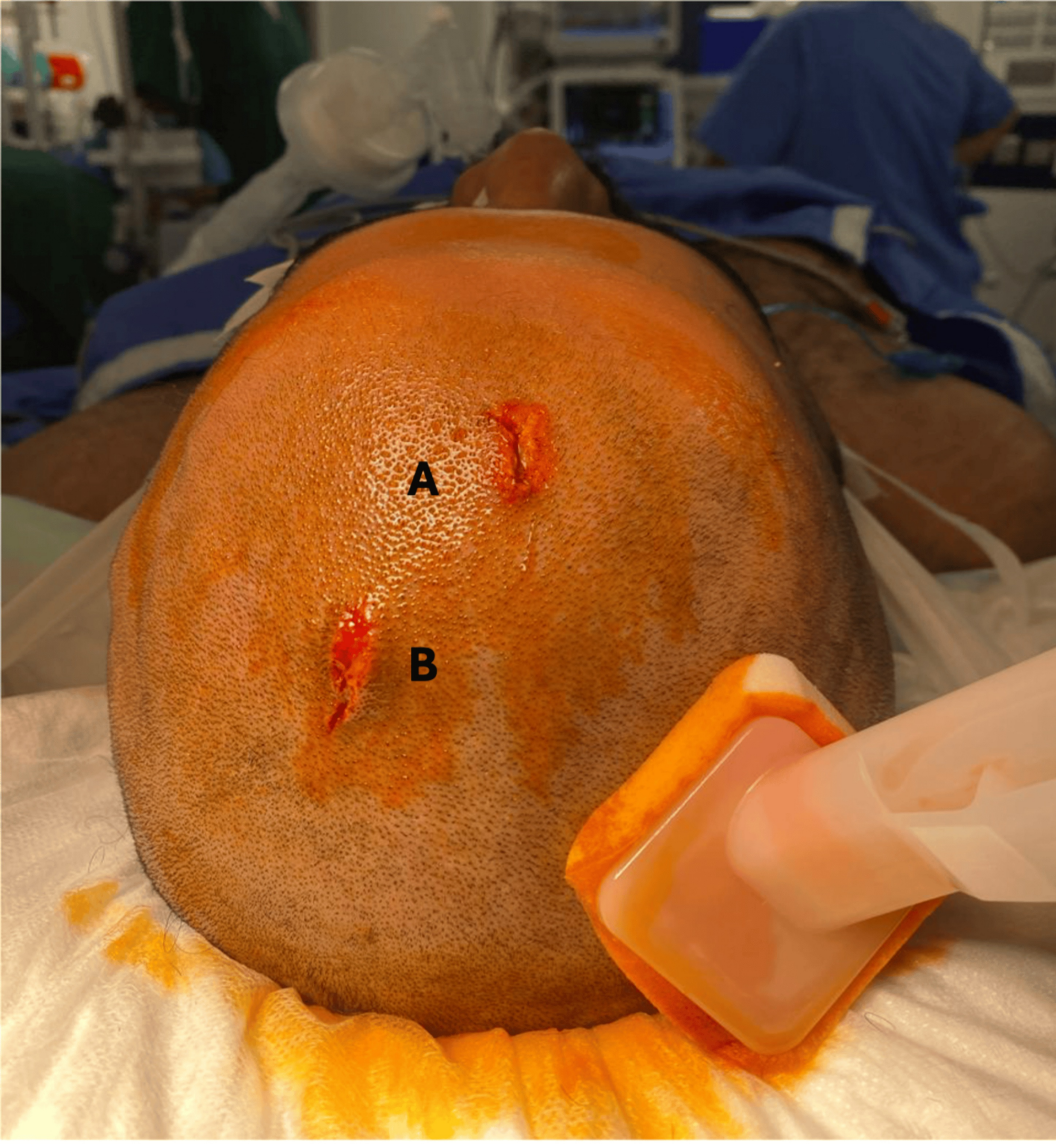
Gross entry wound with an abrasion rim (A) and exit wound (B)

The patient reported a history of type 2 diabetes mellitus and denied any smoking, alcohol, or illicit drug use. According to the patient, he was not the intended target of the shooting incident. Neurological examination revealed equal and reactive pupils bilaterally, full motor strength in all limbs, and normal cranial nerve function. Wound characteristics were consistent with a ricochet injury. The entrance wound demonstrated an abrasion rim with inverted edges (Figure 1).

**Figure 2:**
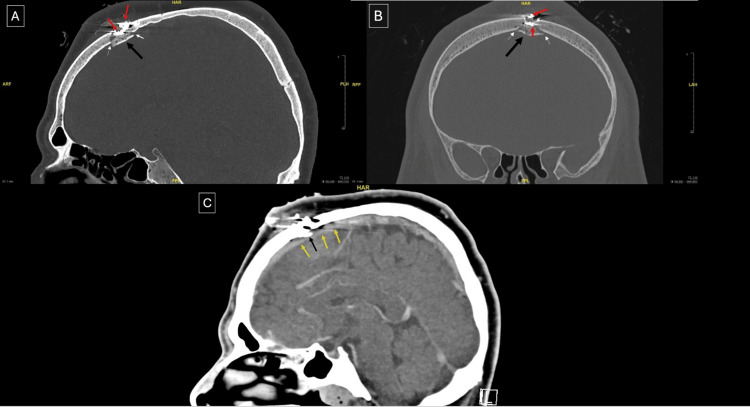
Preoperative non-contrast head CT demonstrating a bony island formed by the fracture (black arrow), with fracture margins indicated by white arrows. Red arrows denote bullet fragments adherent to the bony island. Yellow arrows indicate the SSS in close proximity to the bony island. (A) Sagittal section, bone window. (B) Coronal section, bone window. (C) Sagittal section, brain window CT: computed tomography, SSS: superior sagittal sinus

**Figure 3:**
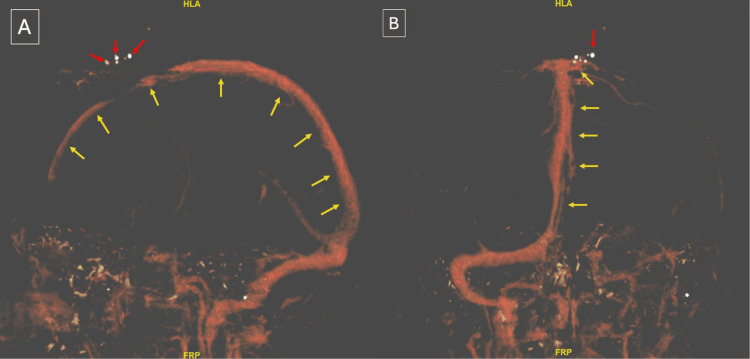
Preoperative head CT venography demonstrating the SSS (yellow arrows) and bullet fragments (red arrows). (A) Anteroposterior view. (B) Caudocranial view CT: computed tomography, SSS: superior sagittal sinus

Laboratory investigations revealed an elevated glycated hemoglobin level of 8.3% and a random blood glucose level of 261 mg/dL. All other laboratory findings were within normal limits. A non-contrast head computed tomography (CT) followed by CT angiogram and venogram was performed to assess the injury and ensure the patency of the superior sagittal sinus (SSS). Imaging showed a comminuted fracture of the left frontal bone with focal left parasagittal hyperdensity representing a small hematoma (Figure 2). The intracranial venous sinuses were well opacified, with no evidence of extravasation (Figure 3). The patient was subsequently admitted to the surgical intensive care unit.

After 48 hours, an operating room became available, and the patient was transferred for surgical debridement. Debridement of the skin and subcutaneous tissue was performed, and superficial bullet fragments were removed. Bullet fragments that were deeply adherent to the depressed bone were left in situ. The decision not to elevate the bone fragment is analyzed in detail in the surgical management section. The patient was extubated postoperatively and monitored in the ICU for 24 hours, remaining neurologically intact and hemodynamically stable.

**Figure 4:**
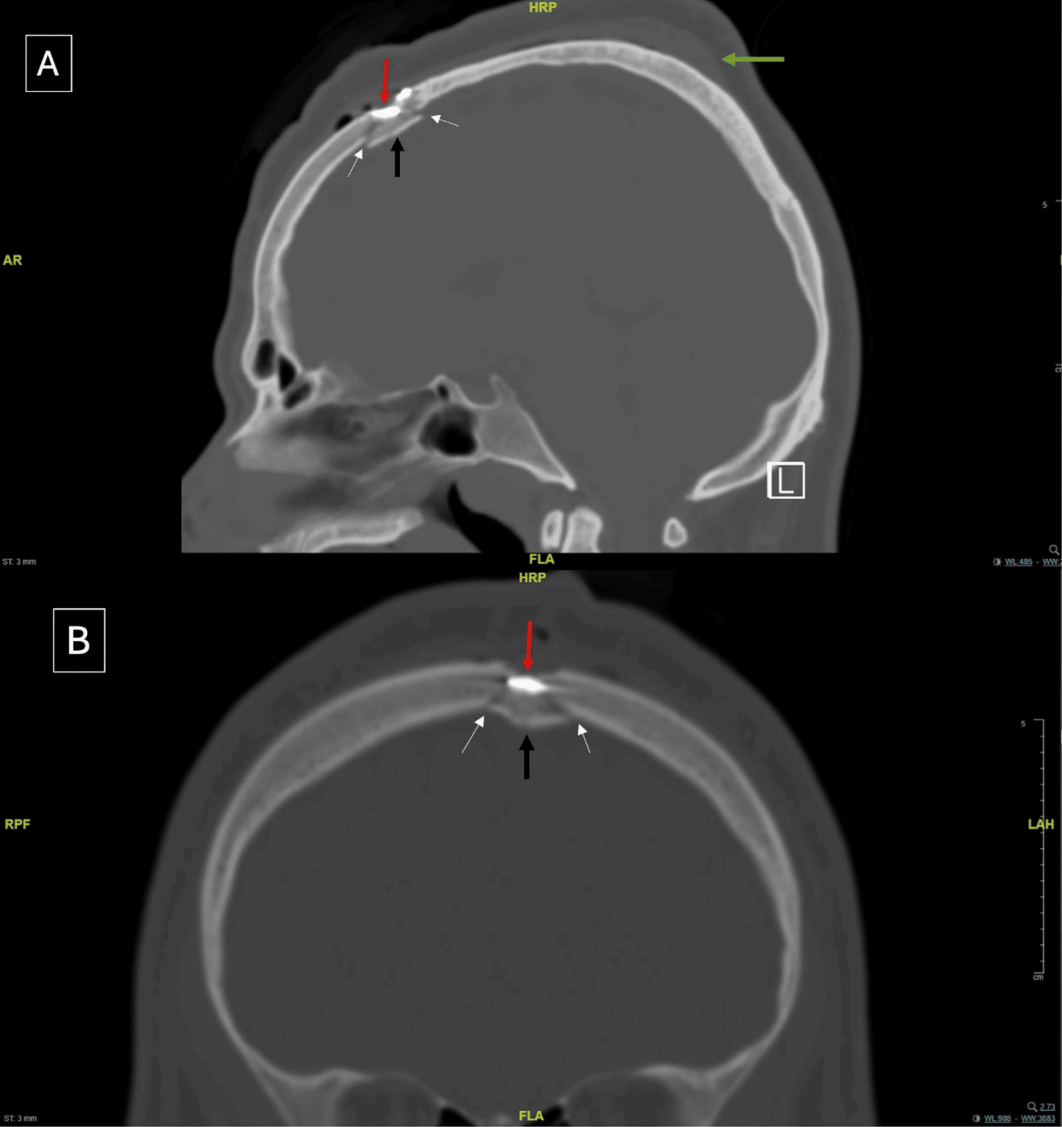
Postoperative non-contrast head CT demonstrating a subgaleal hematoma (green arrow), bullet fragments (red arrow), the bony island (black arrow), and fracture lines (white arrows). (A) Sagittal view. (B) Coronal view CT: computed tomography

On the following day, the patient was transferred to the regular ward and began mobilizing without complaint. A follow-up CT scan revealed residual metallic density in the left parasagittal region with an associated depressed fracture, as previously noted. Dual extra-axial bleeding at the trauma site and a subgaleal hematoma were also present. There was no evidence of intra-axial hemorrhage or ischemia (Figure 4). The patient remained under observation in the ward for five days with an uneventful hospital course. On day 6, he showed clinical improvement and was discharged in stable condition on cefuroxime 500 mg twice daily. He was scheduled for a follow-up in the neurosurgery clinic in 2 weeks but missed his appointment.

## Discussion

Management

Neurosurgical management of GSWH remains a subject of debate, and current evidence is largely derived from retrospective and observational studies. Surgical intervention is generally considered futile in patients with minimal neurological function, such as those with fixed pupils or decorticate/decerebrate posturing [10]. Among operable cases, the extent of debridement is controversial. Some neurosurgeons advocate for aggressive removal of all bone and metallic fragments, whereas others support limited tissue debridement with maximal preservation of cerebral tissue [11]. Surgical goals typically include hematoma evacuation, hemostasis, removal of accessible bone fragments, and secure watertight dural closure, often requiring allograft or artificial materials to prevent cerebrospinal fluid (CSF) leak and associated infections [10,12]. Identification of entry and exit sites is also essential for medicolegal analysis. Postoperative elevation of intracranial pressure (ICP) may indicate that more extensive debridement was required intraoperatively [10].

In the present case, initial non-contrast CT imaging demonstrated a high-velocity penetrating injury with an entry track at the mid/left parasagittal frontal bone. Multiple subcutaneous metallic densities representing fragmented foreign bodies were noted, accompanied by a small subgaleal hematoma with an underlying comminuted fracture of the mid/left parasagittal frontal bone. There was mild inward displacement of a small bony fragment and adjacent extra-axial hemorrhage in the left frontal parasagittal region. CT venography showed no extravasation or filling defect, and the venous sinuses were well opacified.

Intraoperatively, a 3-cm linear incision was made connecting the entry and exit wounds, with excision of burned skin, followed by debridement of the skin and subcutaneous tissue. A diamond drill was used to shave superficially adherent bullet fragments to ensure the removal of all accessible fragments as far as feasible. Deeper bullet fragments were firmly adherent to the bony island formed by the fracture. This bony island was in direct contact with the superior sagittal sinus (SSS). Therefore, the remaining fragments were intentionally left in situ to avoid excessive manipulation and potential injury to the SSS. Finally, the wound was irrigated with saline, hydrogen peroxide, and povidone-iodine. Layered closure was achieved using Vicryl and Prolene sutures. Our approach aligns with the ongoing trend favoring avoidance of unnecessary bone elevation and removal of deeply embedded fragments to minimize iatrogenic neurological damage and morbidity [11,13,14].

Conventionally, skull fractures overlying the SSS are managed conservatively, as fracture elevation carries a risk of bleeding [15]. However, there are limited reports in the literature in which avoidance of bone elevation constituted the definitive management for patients with penetrating head trauma resulting in depressed skull fractures overlying the SSS. Most reported cases involve significant SSS rupture or compression, thereby necessitating extensive surgical intervention.

One similar case was reported by Zoerner et al. [16], describing a 14-year-old boy with a depressed skull fracture secondary to non-penetrating head trauma. Initial imaging showed a large subgaleal hematoma with disruption and thrombosis of the SSS; however, the patient was asymptomatic. Given the risk of hemorrhage, non-surgical management was decided, and the depressed parietal bone fragment was left in place to preserve the tamponade effect. The patient achieved full recovery within one week and was discharged home.

In contrast, Birk et al. [17] reported a case of a 25-year-old female patient with a depressed skull fracture secondary to a GSWH. Unlike our case, the patient showed symptoms of venous outflow compromise; therefore, the surgeon opted for bilateral craniectomy. The patient recovered with full resolution of symptoms. This case underscores that, when evidence of venous compromise is present, emergent surgical decompression should be considered.

Complications

GSWH is associated with a broad spectrum of acute and delayed complications. Vascular injuries, including arteriovenous fistulas, traumatic aneurysms, arterial thrombosis, and dissection, occur in 38-50% of patients with penetrating traumatic brain injury. While CT angiography has demonstrated high sensitivity (100%) in detecting traumatic aneurysms in civilian penetrating brain injuries (cvPBI), its overall sensitivity in identifying arterial cvPBI is lower (72.7%). Thus, despite resource constraints in the acute setting, conventional angiography remains the gold standard for diagnosing arterial injury [18].

Infectious complications are precipitated by retained bullet fragments, necrotic tissue, bony debris, and insufficient debridement. Increased infection risk is associated with residual metallic or osseous fragments on postoperative CT, trajectories involving the paranasal sinuses or oral cavity, and lower GCS or Glasgow Outcome Scale scores [19].

A 2022 study examining pediatric survivors of GSWH found infection to be the most common complication, with pneumonia, meningitis, and sepsis occurring in 21%, 16%, and 11% of cases, respectively. Additional complications included sympathetic storming and cerebral salt-wasting (26%), CSF leaks, seizures, diabetes insipidus, facial palsy, pseudoaneurysms, and unexplained fevers (each 11%) [20].

Prognosis and clinical outcomes

A 2018 meta-analysis of 1,774 civilian GSWH cases identified several factors associated with increased mortality. These included age >40 years, ICP >20 mmHg, bilaterally fixed and dilated pupils, GCS <9, suicidal intent, trajectories involving bihemispheric, multilobar, or transventricular paths, and lack of operative intervention [21].

Similarly, a retrospective cross-sectional study reported worse outcomes in patients presenting with a GCS of 3-7. Survivors were more commonly found to have at least one reactive pupil, although age did not correlate significantly with outcome. CT findings associated with mortality included intraventricular hemorrhage and space-occupying lesions. Trajectories in non-survivors often involved a defined "potentially lethal zone," bounded superiorly by the frontal horns of the lateral ventricles, posteriorly by the brainstem, inferiorly by the dorsum sellae, anteriorly by the basal frontal region, and laterally by the temporal horns of the lateral ventricles. Injuries occurring within this zone, and approximately 25.3 mm from the circle of Willis, were associated with a higher mortality rate [22].

## Conclusions

GSWH carries significant implications for public safety, morbidity, and mortality. This case illustrates a young patient who survived a ricochet cranial gunshot injury without neurological deficits, an uncommon and favorable outcome given the complexity and unpredictability of ricochet trajectories. Although ricochet injuries generally have reduced penetrating capacity, involvement of critical structures such as the SSS can still cause life-threatening damage. Optimal management requires coordinated care across emergency medicine, neurosurgery, radiology, and critical care. Reporting similar cases can help refine best-practice guidelines for evaluating and managing firearm-related injuries.
